# Structural and sequencing analysis of local target DNA recognition by MLV integrase

**DOI:** 10.1093/nar/gkv410

**Published:** 2015-05-12

**Authors:** Sriram Aiyer, Paolo Rossi, Nirav Malani, William M. Schneider, Ashwin Chandar, Frederic D. Bushman, Gaetano T. Montelione, Monica J. Roth

**Affiliations:** 1 Department of Pharmacology, Robert Wood Johnson Medical School, Rutgers University, Piscataway, NJ 08854, USA; 2 Center for Advanced Biotechnology and Medicine, Department of Molecular Biology and Biochemistry, and Northeast Structural Genomics Consortium (NESG), Rutgers University, Piscataway, NJ 08854, USA; 3 Department of Microbiology, Perelman School of Medicine, University of Pennsylvania, Philadelphia, PA 19104, USA; 4 Department of Biochemistry, Robert Wood Johnson Medical School, UMDNJ, Piscataway, NJ 08854, USA; 5 Department of Biochemistry and Molecular Biology, Robert Wood Johnson Medical School, Rutgers University, Piscataway, NJ 08854, USA

## Abstract

Target-site selection by retroviral integrase (IN) proteins profoundly affects viral pathogenesis. We describe the solution nuclear magnetic resonance structure of the Moloney murine leukemia virus IN (M-MLV) C-terminal domain (CTD) and a structural homology model of the catalytic core domain (CCD). In solution, the isolated MLV IN CTD adopts an SH3 domain fold flanked by a C-terminal unstructured tail. We generated a concordant MLV IN CCD structural model using SWISS-MODEL, MMM-tree and I-TASSER. Using the X-ray crystal structure of the prototype foamy virus IN target capture complex together with our MLV domain structures, residues within the CCD α2 helical region and the CTD β1-β2 loop were predicted to bind target DNA. The role of these residues was analyzed *in vivo* through point mutants and motif interchanges. Viable viruses with substitutions at the IN CCD α2 helical region and the CTD β1-β2 loop were tested for effects on integration target site selection. Next-generation sequencing and analysis of integration target sequences indicate that the CCD α2 helical region, in particular P187, interacts with the sequences distal to the scissile bonds whereas the CTD β1-β2 loop binds to residues proximal to it. These findings validate our structural model and disclose IN-DNA interactions relevant to target site selection.

## INTRODUCTION

Retroviral integrase (IN) proteins mediate an indispensable step in the retrovirus replication, the irreversible integration of viral cDNA into the host genome. The first step in integration involves removal of the terminal dinucleotide of the viral long terminal repeats (LTRs) produced by reverse transcription, exposing the 3′OH group of the invariant CA dinucleotide. The processed LTR DNA ends are then inserted into the host genome through an energy-independent transesterification step known as the strand transfer reaction (1). For murine leukemia virus (MLV), integration results in a target site duplication (TSD) of 4 bp, 3′ to the invariant viral CA dinucleotide. At the chromosomal level, MLV integration preferentially occurs at transcription start sites and CpG islands (2). More recently it has been shown that strong enhancers are the predominant targets of MLV integration (3,4).

The MLV IN^1–408^ belongs to the polynucleotidyl phosphotransferase superfamily of enzymes that includes RNase H, Mu transposase and Tn5 transposase (5). Retroviral IN proteins contain three functional domains; an N-terminal zinc binding domain (NTD), the catalytic core domain (CCD) encoding an invariant catalytic D, D (35) E triad, and a C-terminal domain (CTD). Additionally, both the prototype foamy virus (PFV) IN and the MLV IN encode an N-terminal extension domain (NED) within the NTD that is not conserved in the human immunodeficiency virus -1 (HIV-1) IN (6). Of the three IN domains, the CTD has the least sequence conservation among all retroviral INs (7,8), but structural analysis of the isolated HIV-1 IN CTD (9,10) and the Rous sarcoma virus (RSV) IN CTD in the two-domain structure has indicated that they adopt an SH3 fold (11). Recent studies have indicated that minimally the CTD is required for interaction with the bromo- and extra terminal (BET) family of host cell proteins that guide integration targeting by tethering pre-integration complexes near transcription start sites (12–14).

Crystal structures of the full-length PFV IN tetramer in complex with both viral (LTR) and target DNA (tDNA) have been solved (6,15), providing key background for studies reported here. These structures are distinct from prior molecular models of IN tetramers and from single- or two-domain IN structures. In the complex, the inner dimer of PFV IN, required for functional 3′ processing and strand-transfer, displays complex intermolecular interactions among multiple domains; however, the outer IN dimers interact solely through the CCD. The NTD and CTD domains of the outer IN molecules are not resolved in the PFV structures (6,15) although their organization has been predicted using small angle X-ray scattering (SAXS) analysis (16). Based on the PFV intasome (6) structures, residues within the CCD and CTD that are important for binding tDNA have been identified. Within the CCD, the α2 helix has previously been implicated in tDNA binding (17). Additional determinants for tDNA binding within the PFV CTD localize within the β1-β2 loop and the β4 strand (15). In particular, the PFV IN R329 interacts with multiple bases within the tDNA and induces a bent confirmation. In HIV-1 IN, a serine residue (S119) mediates base contacts within the CCD in a manner similar to the homologous PFV IN A188 (18). Similarly, in the HIV-1 IN CTD, the function of PFV IN R329 is mediated by the homologous R231, although other residues in the β1-β2 loop also have the potential to interact with tDNA (18). Furthermore, it was established that HIV-1 IN shows a propensity to target flexible dinucleotide steps in the central TSD region flanked by rigid dinucleotide steps.

Here we describe the production, purification, and nuclear magnetic resonance (NMR) solution structure of the MLV IN CTD^329–408^. This includes collection and processing of the NMR data, as well as the determination, validation and interpretation of the solution NMR structure. This structure, deposited into the Protein Data Bank (2013, PDB ID 2M9U), has been instrumental in defining the interactions of the IN CTD with the Brd3 ET domain in an independent study (12). By structural comparison of this MLV IN CTD with the PFV IN CTD and homology modeling of the MLV IN CCD, regions predicted to be involved in tDNA binding could be identified. A comprehensive characterization of mutants within these putative target binding sites in full-length proviral genomes, including chimeras of PFV/MLV or HIV/MLV, was performed using a tissue culture system for viral replication. The consequence of these mutants in the context of the CCD and CTD structure was analyzed by deep sequencing of viral integration sites allowing analysis of the mechanisms responsible for local sequence preference and recognition of the flexibility of tDNA in the vicinity of integration sites.

## MATERIALS AND METHODS

### Cloning, expression and purification of the CTD of MLV IN

The CTD (residues 329–408) of MLV IN was polymerase chain reaction (PCR) amplified from the pNCA-C plasmid (19), an infectious clone of MLV. PCR amplification utilized forward primer 5′ GGAATTCCATATGGTGGGCGATACCGTGTGGGTG 3′ and reverse primer 5′ CGGGATCCCGTCACGGCGCTTCGCG 3′, with the underlined sequences indicating NdeI and BamHI sites respectively, introduced for cloning into the pET15_NESG vector (20,21). The vector contains an in-frame N-terminal non-cleavable hexahistidine tag, before the NdeI sequence (NESG Construct ID: OR41A-15.1). Protein expression and purification from the pET-based construct was performed as previously described (20,21) with the following modifications: protein expression was induced with 1 mM isopropyl β-D-1-thiogalactopyranoside (IPTG) at 17°C for 25 h. Induction was carried out in MJ9 media (22) in the presence of either ^15^N-labeled ammonium chloride or ^15^N ammonium chloride plus ^13^C glucose. Following Ni-NTA resin purification (Qiagen) as per manufacturer's instructions, fractions eluted in 400 mM imidazole were pooled and concentrated to a volume of less than 250 μl using an Amicon Ultracel-3K centrifugal filter unit (Millipore). The concentrated protein fraction was then injected into an AKTA FPLC and resolved on a Superdex 75 gel filtration column (GE Healthcare) in 20 mM MES pH 6.5, 100 mM NaCl and 50 mM potassium glutamate (KC_5_H_8_NO_4_). The eluted fractions were then pooled and concentrated using an Amicon Ultracel-3K centrifugal filter unit (Millipore). Final protein yields ranged from 16 to 20 mg/l of bacterial culture. Homogeneity (> 97%) was validated by sodium dodecyl sulphate-polyacrylamide gel electrophoresis, and the protein identity was verified by matrix assisted laser desorption/ionization-time of flight (MALDI-TOF) mass spectrometry. The ^15^N and ^15^N plus ^13^C labeled protein samples were then concentrated to approximately 8–10 mg/ml in the same buffer for NMR analysis. All isotopes were purchased from Cambridge Isotopes Laboratories.

### Structure determination of MLV IN CTD

The NESG Target ID for the MLV CTD is OR41A with the NESG Construct ID OR41A-15.1. Isotopically enriched samples for NMR spectroscopy of NESG construct OR41A-15.1 were prepared and analyzed using standard published protocols (20,23). Samples were prepared for NMR studies at protein concentration of 0.5 mM in 10% ^2^H_2_O, 50 mM DSS, 100 mM NaCl, 50 mM potassium glutamate, 20 mM MES pH 6.5. All spectra were recorded using a Bruker Avance 800 MHz instrument. Sample temperatures were maintained at 25°C. NMR data were processed using NMRpipe (24) and SPARKY (T. D. Goddard and D. G. Kneller, SPARKY 3, University of California, San Francisco). Complete ^1^H, ^13^C and ^15^N resonance assignments for OR41A were carried out using standard triple resonance experiments (25) and 3D structure determination was carried using standard methods (26,27). Analysis of nuclear Overhauser effect spectroscopy (NOESY) cross peaks and structure generation calculations were performed using the program *Cyana* (28,29) and constrained energy refinement was carried out using the program *CNS* in explicit solvent (30). Validation of the resulting ensemble of structures was performed using the Protein Structure Validation Server (PSVS) (31,32) and Recall, Precision, F-measure (RPF) analysis of the agreement between structural models and NOESY peak list data (33). ‘PROMALS3D’ was used for generating structure-based sequence alignments of the PFV, HIV and MLV CTD structures (34,35).

### Transient transfection of the proviral clone in D17pJET cells

Mutations were introduced into the pNCA-C vector backbone using overlapping PCR and confirmed by traditional Sanger sequencing. A list of oligonucleotides used for introducing the mutations will be provided upon request. 10^5^ canine D17pJET cells expressing the murine cationic amino acid transporter (MCAT) receptor (36) were cultured in a 60 mm dish with 5 ml of Dulbecco's modified Eagle's medium (Gibco), 10% (vol/vol) fetal bovine serum (Atlanta Biologicals) and 1% (vol/vol) antimycotic/antibiotic (Gibco). The next day, 250 ng of the proviral clone of MLV pNCA-C (19) and the various IN mutations in the pNCA-C backbone were transfected with diethylaminoethyl (DEAE)-dextran as previously described (37) and cultures were maintained for at least 40 days. Supernatants were collected at confluence and viral replication was assessed by the presence of reverse transcriptase in the culture media (38).

### Homology modeling of CCD IN

An automated approach was taken for obtaining the model of MLV CCD IN using the SWISS-MODEL (39–41), MMM-tree (42) and I-TASSER (43–45) servers. For all servers, the PFV IN CCD target capture complex (PDB ID:3OS1) was selected as template since we were interested in obtaining information for residues in MLV CCD IN that could be involved in tDNA binding. The resulting homology models were validated using PSVS (31,32,46). Validation was performed on all residues (residues 117–271) of the MLV CCD models as well as the PFV CCD structure as a validation control. The validated model structures were overlaid onto the PFV CCD IN and HIV CCD IN (PDB ID: 1EX4) structures. In order to generate structure-based sequence alignment for identifying tDNA binding residues the PFV CCD IN, HIV CCD IN and the SWISS-MODEL MLV CCD IN were aligned using the ‘PROMALS3D’ server (34,35).

All figures were generated using PyMOL and all overlays were generated using the super_all script within PyMOL (The PyMOL Molecular Graphics System, Version 1.2r3pre, Schrödinger, LLC.)(47).

### 454 pyrosequencing of viral integrants

Transient transfection of HEK293mCAT cells with pNCA-C variants (19), passage of virus and sequencing of viral integrants were performed exactly as described before (12). Sequences were deposited in the sequence read archive (SRA) with accession number SRP04876.

### Western blotting

Western blotting was performed as described previously (48,49). The polyvinyl difluoride (PVDF) membrane blot that was probed for capsid (CA) (goat anti-capsid 75S-287, National Cancer Institute Repository) was stripped in 50 mM Tris-HCl pH 8.3, 50 mM DTT, 1 mM EDTA, 8 M guanidine-HCl with gentle rocking at room temperature for 30 min followed by washing with tris-buffered saline and Tween-20 buffer (TBST). A combination of four different IN antisera was used to probe the same blot: Rabbit 1 bleed 6, Rabbit 2 bleed 5, Rabbit 3 bleed 6 and Rabbit 4 bleed 5 (50). Quantification of CA blot was performed using the Image Studio Lite software (LI-COR Biosciences).

## RESULTS

### Structure-based design for stable expression of an MLV IN CTD construct

**Figure 1.**
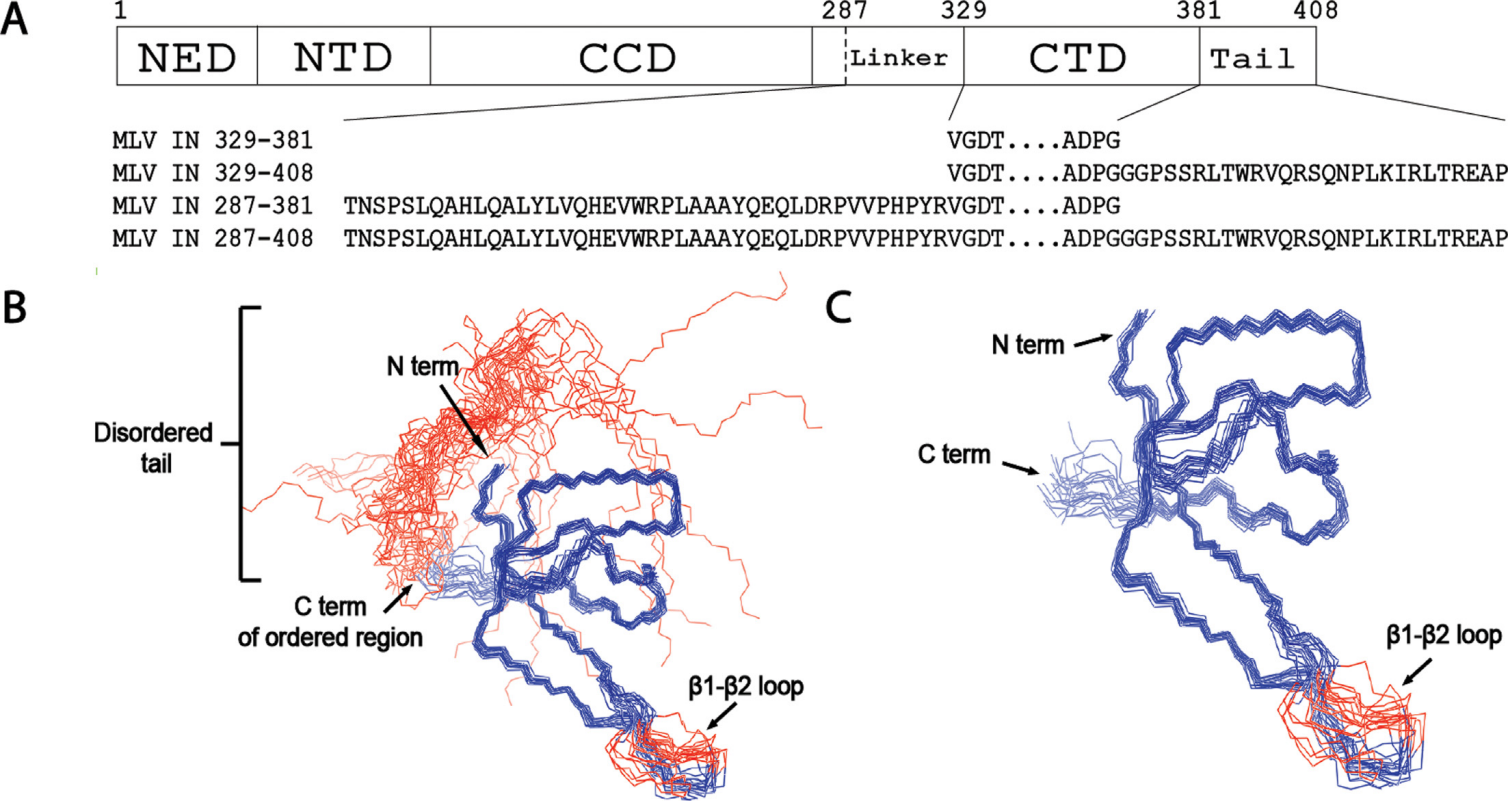
MLV IN CTD. (**A**) Schematic of MLV IN. The domain boundaries NED, NTD, CCD and CTD are indicated with solid vertical lines. Residue 287, previously defined as the CCD/CTD boundary (51), is indicated with a dashed line. Residues 382–408 constitute the C-terminal tail. The sequences of the four MLV IN CTD constructs tested are indicated. (**B**) The Cα backbone trace of IN 329–408. The backbone (N, Cα and C′) atomic coordinates for the 20 lowest energy conformers, which represent the solution NMR structure, are shown with blue lines. The N- and the C-termini, along with the β1-β2 loop, are labeled. The apparently flexible disordered C-terminal tail region (residues 382–408) is represented with red lines. A stretch of three amino-acid residues within the β1-β2 loop (residues 339–341) is not well defined in the atomic coordinates and appears to be disordered. The structure of the hexahistidine tag is also not well defined and has not been displayed. (**C**) The ordered Cα backbone trace of 20 NMR ensemble structures of IN 329–381 (blue) without the apparently flexible disordered C-terminal tail region (residues 382–408).

Limited proteolysis experiments as well as linker scanning studies of MLV IN suggested that IN residue 287 defined the boundary between the CCD and CTD (51). However, purification of IN^287–408^ was largely unsuccessful, with most of the protein forming insoluble inclusion bodies when expressed in a variety of bacterial expression conditions. Truncation of the C-terminal 27 residues to yield IN^287–381^ did not improve solubility, though a similar truncation of the C-terminal amino acids was employed for elucidating the HIV-1 IN CTD structure (10). The X-ray structure of PFV IN revealed the homologous MLV IN region between amino acids 287 and 329 to be a large extended linker region. The overall size similarity of the PFV IN and MLV IN prompted us to redefine the boundary of the CTD as residue 329, which in PFV IN precedes the first beta strand of the SH3 fold region by a few residues (Figure 1A). The solubility and expression of the modified MLV IN CTD^329–381^ and MLV IN CTD^329–408^ were markedly improved, and as a result, we were able to routinely purify and concentrate a non-cleavable hexahistidine tagged construct to 8–10 mg/ml. The purified MLV IN CTD^329–408^ construct migrates at the predicted molecular mass of approximately 10 kDa by electrophoresis (Supplementary Figure S1A) and was used for solution NMR structure determination.

For NMR structural studies, the complexity of the analysis is dependent on the multimeric state of the protein. The HIV-1 IN CTD DNA binding domain was a dimer in the solution structure (9,10). Analysis of the MLV IN CTD^329–408^ construct on Superdex 75 size exclusion column indicated the protein eluted as a monomer of estimated molecular weight of 11.02 kDa, based on the plot of the protein standards of Kav^1/3^ versus Molecular weight^0.555^ (Supplementary Figure S1B). The variation from the predicted molecular mass of the protein (10.03 kDa) might reflect the presence of the long unstructured C-terminal tail (residues 382–408; Figure 1B). The monomeric state of the MLV IN CTD^329–408^ was further confirmed by NMR where the *T*_1_/*T*_2_ (CPMG) measurement (*T*_1_ = 717 ms, *T*_2_ = 100 ms at 25°C) yielded a rotational correlation time of 6.0 ns, which corresponds to the ∼10 kDa predicted molecular mass of the protein (Supplementary Figure S1C) (52).

### MLV IN CTD NMR structure

The structure of the MLV IN CTD was determined by solution NMR spectroscopy. The superimposition of the well-defined regions of 20 conformers that form the ensemble of structures is displayed in Figure 1B and C along with a stereo view (Supplementary Figure S1D). Structure quality statistics for this ensemble are displayed in Table 1. Backbone chemical shift data and the absence of NOESY cross peaks indicate a disordered structure in the C-terminal region from residue G382 to the C-terminus (Figure 1B and Supplementary Figure S2). The final ensemble of 20 structures (Figure 1B and C) was deposited in the Protein Data Bank (PDB ID: 2M9U) and BioMagResDB (BMRB ID: 19299).

**Table 1.** Summary of structural statistics^a^

	MLV IN CTD	
Completeness of resonance assignments^b^:		
Backbone (%)	84.6	
Side chain (%)	61.2	
Aromatic (%)	100	
Stereospecific methyl (%)	100	
Conformationally restricting constraints:		
Distance constraints		
Total	393	
Intraresidue (*i* = *j*)	85	
Sequential (|*i* − *j*| = 1)	111	
Medium range (1 < |*i* − *j*| < 5)	44	
Long range (|*i* − *j*| ≥ 5)	157	
Dihedral angle constraints	147	
Hydrogen bond constraints	44	
No. of constraints per residue	7.6	
No. of long range constraints per residue	2.6	
Residual constraint violations^c^:		
Average no. of distance violations per structure:		
0.1–0.2 Å	1.7	
0.2–0.5 Å	0.05	
> 0.5 Å	0	
Average no. of dihedral angle violations per structure:		
1–10°	18.4	
> 10°	0	
Model quality^c,d,e^:		
RMSD backbone atoms (Å)	0.6	
RMSD heavy atoms (Å)	0.9	
RMSD bond lengths (Å)	0.018	
RMSD bond angles (°)	1.2	
MolProbity Ramachandran statistics:		
Most favored regions (%)	94.3	
Allowed regions (%)	5.5	
Disallowed regions (%)	0.2	
Global quality scores^c^ (Raw / *Z*-score):		
Verify3D	0.28	-2.89
Prosa II	0.12	-2.19
Procheck (phi-psi)	-0.82	-2.91
Procheck (all)	-0.45	-2.66
Molprobity clash score	13.54	-0.8
RPF Scores:		
Recall / Precision	0.888	0.968
F-measure / DP-score	0.926	0.821
Model contents^d^:		
Ordered residue ranges^c^	329–338, 342–381	
BMRB accession code:	19299	
PDB accession code:	2M9U	

^a^Structural statistics were computed for the ensemble of 20 deposited structures of lowest energy.

^b^Computed using AVS software (32) from the expected number of peaks, excluding highly exchangeable protons (N-terminal, Lys and Arg amino groups, hydroxyls of Ser, Thr, Tyr), carboxyls of Asp and Glue, non-protonated aromatic carbons and the N-terminal 6-His tag.

^c^Calculated using PSVS 1.4 program (31). Average distance violations were calculated using the sum over *r*^−6^. The abbreviation RMSD represents root-mean-square deviation.

^d^Based on ordered residue ranges [S(phi) + S(psi) > 1.8]. Ordered affinity tag sequence is not included.

^e^RPF scores (33) reflecting the goodness-of-fit of the final ensemble of structures to the NOESY peaklists (3D-^13^C,^15^N edited NOESY in H_2_O, 3D-^13^C-NOESY in ^2^H_2_O calculated with same tolerances as for CYANA-3.0 automated NOE assignment and structure calculation run (0.05 ppm ^1^H ind., 0.4 ppm ^13^C,^15^N and 0.03 ppm obs. ^1^H). All residues with chemical shift assignment are included in the analysis.

**Figure 2.**
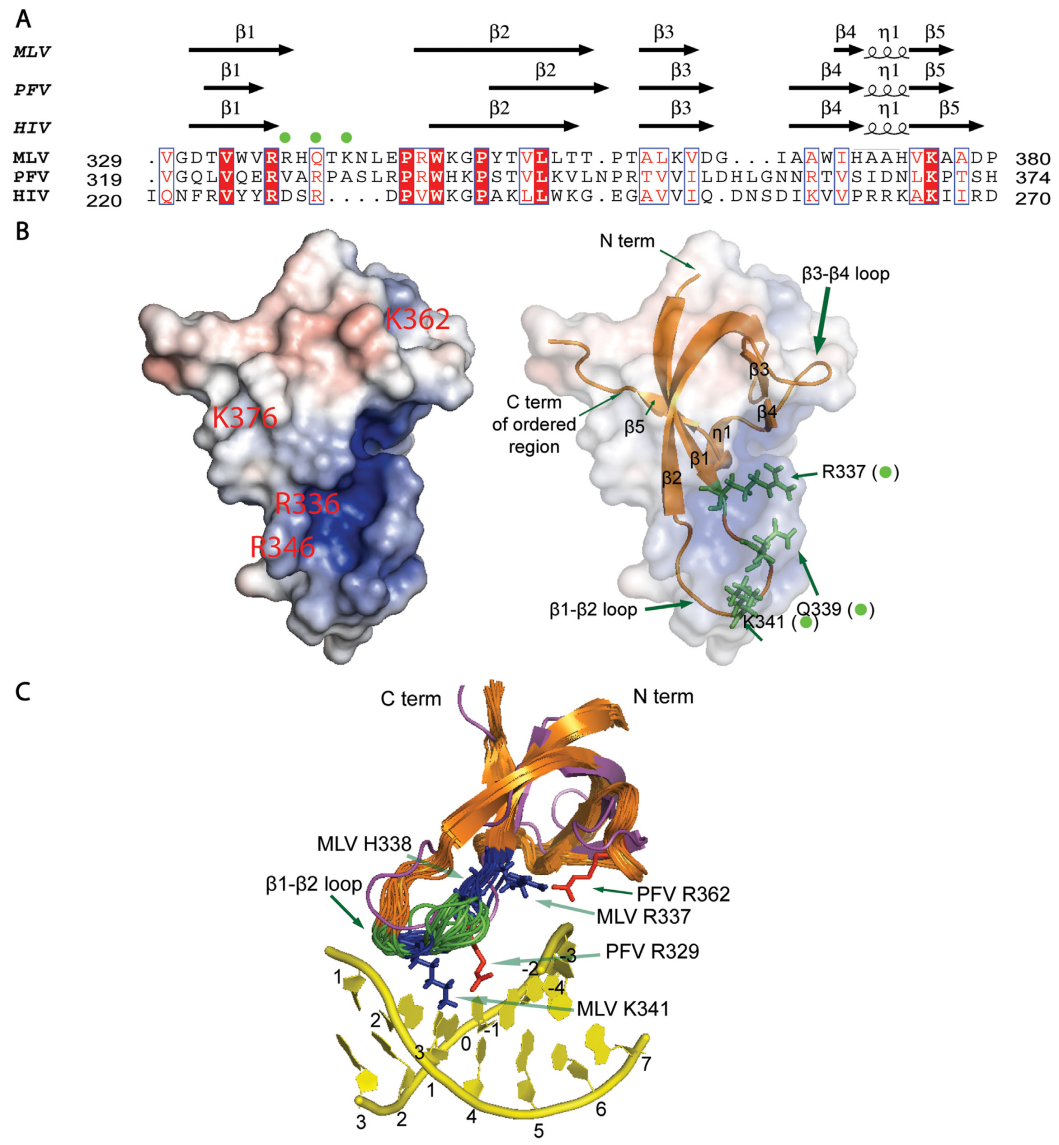
CTD structure and modeling of tDNA binding sites. (**A**) PROMALS3D structure-based sequence alignment for the MLV CTD, PFV CTD (PDB ID: 3OS1) and HIV CTD (PDB ID: 1IHV) is displayed using the ESPRIPT server output (35). All default parameters were used for the alignment. The given alignment is constrained to a prior PROMALS3D alignment of the MLV and PFV CTDs alone. Residues 329–380 of the MLV CTD, 319–374 of the PFV CTD and 220–270 of the HIV CTD are displayed. The three predicted tDNA binding residues R337, Q339 and K341 are marked in the alignment with green dots. (**B**) Electrostatic surface map with two different transparent sphere illustrations to depict the organization of the SH3 fold and predicted tDNA binding residues. Green sticks indicate side chains of MLV IN residues R337, Q339 and K341 (also marked with green dots). Images were generated using the APBS plugin (47) in PyMOL (The PyMOL Molecular Graphics System, Version 1.2r3pre, Schrödinger, LLC.) with the representative model 1 structure from the ensemble of 20 structures. Fully saturated red and blue colors represent, respectively, negative and positive potentials of ±5 kT at an ionic strength of 0.15 M and at a temperature of 298 K. (**C**) Overlay of the CTD ensemble (orange) with the PFV CTD (magenta) and PFV tDNA (yellow) is shown. PFV CTD tDNA binding resides of R329 and R362 are displayed with their side chains as red sticks. Homologous MLV CTD residues of R337 and K341 have side chains that adopt similar positions and are displayed as blue sticks, while the β1-β2 loop region marked in green and blue represents the six amino acids that were exchanged with their PFV counterparts for generation of chimeric viruses. MLV H338 is also marked in blue and this residue along with MLV K341 was found to be important in combinatorial mutational analysis (See Figure 5).

The C-terminal MLV IN domain folds in the standard SH3 topology with five β-strands comprised of the following residues: β1: 331–337, β2: 346–357, β3: 360–363, β4: 370–371, β5: 375–377 and a 3_10_-helix segment comprising residues 372–374 (Supplementary Figure S1D). The MLV IN CTD β1-β2 loop is smaller in length compared to the corresponding PFV IN CTD domain (PDB ID: 3OS1). A six-residue loop (_364_DGIAAW_369_) separates two relatively short beta strands (strands 3 and 4) within the MLV CTD. This region differs from that of the PFV IN (PDB ID: 3OS1) and HIV-1 IN (PDB ID: 1IHV) CTDs, which possess longer strands separated by five- and four-residue loops, respectively. The PFV IN CTD construct has the largest β1-β2 loop among the reported retroviral CTD IN protein structures, potentially providing conformational flexibility allowing interaction with tDNA (Figure 2A). In the PFV IN CTD, R329 in the β1-β2 loop and R362 in the β4 strand make crucial interactions with the tDNA (15).

The hexahistidine tag, part of the β1-β2 loop (residues 339–341) and the C-terminal tail (residues 382–408) are not well defined in the NMR ensemble and appear to be disordered (Figure 1B and C and Table 1). The disordered C-terminal tail region becomes ordered in the presence of the ET domain of BET proteins (12). Model 1 from the ensemble was identified as the representative structure (i.e. the one most similar to all other structures) (53,54) and was used for calculating the Cα root-mean-square deviation (RMSD) between the MLV IN CTD and the PFV and HIV IN CTDs. Despite low-sequence similarities, using the DaliLite server (55), we find that the MLV IN CTD SH3 domain aligns to within 2.2 Å C RMSD of the HIV IN CTD monomer structure (PDB ID: 1IHV) and to within 2.9 Å C RMSD of the PFV structure (PDB ID: 3OS1). This structure-based sequence alignment highlights residues that are highly conserved or in equivalent positions within the structures of PFV and HIV IN equivalent to PFV and HIV (Figure 2A).

Based on the solvent-exposed electrostatic map (Figure 2B) and the conserved residue Consurf analysis (56) (Supplementary Figure S3A and B), residues R336, H338, R346, K362 and K376 of the MLV IN CTD are conserved among gammaretroviruses and surface exposed. However, only R336 and K376 are conserved with PFV and HIV IN CTDs (Figure 2A). Conversely, R337, Q339 and K341 of the MLV IN CTD are not conserved but located on the important β1-β2 loop and likely to influence tDNA integration sequence specificity (Figure 2B and C, and Supplementary Figure S3A and C).

### Modeling of the CCD of IN

**Figure 3.**
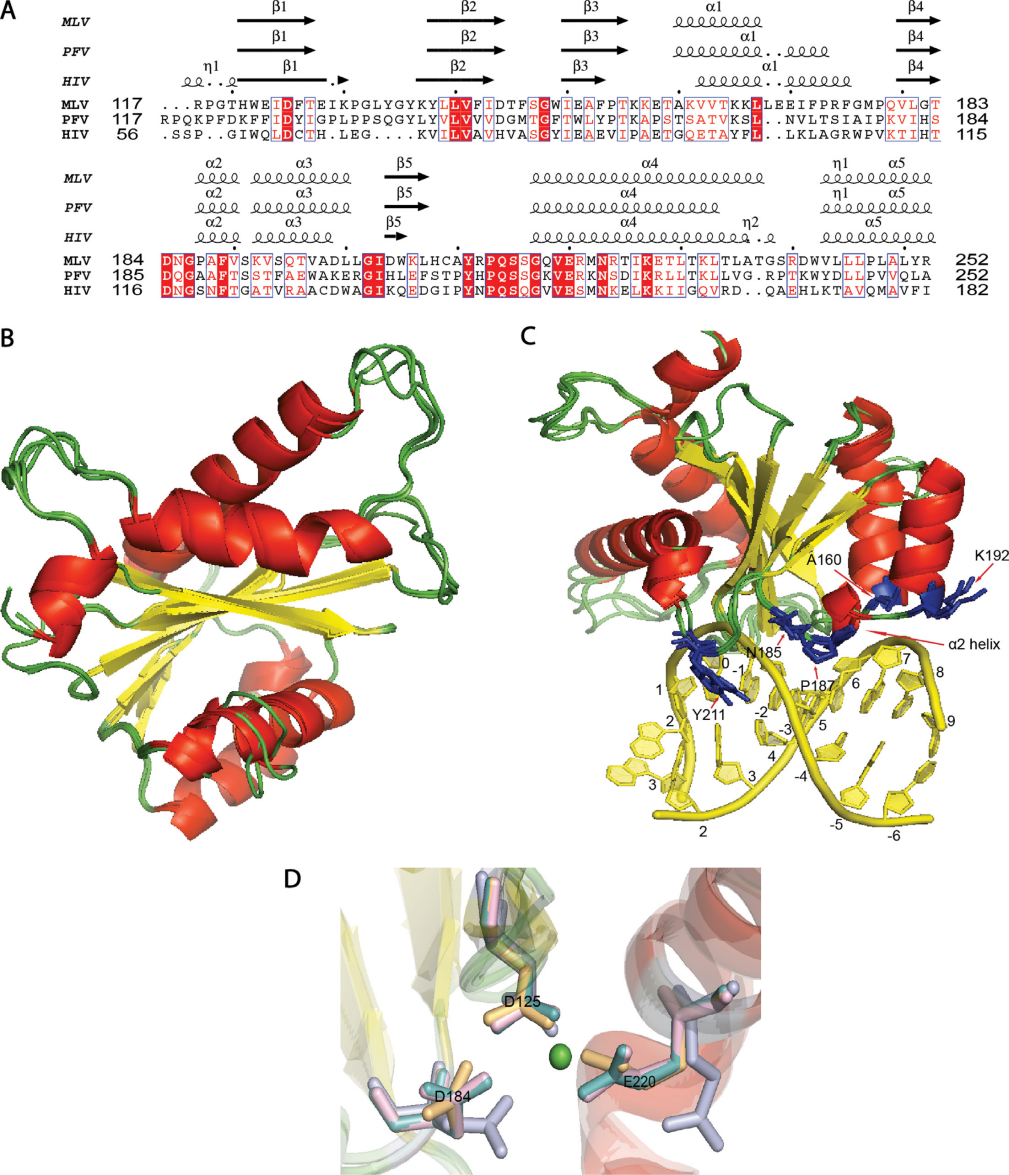
MLV IN CCD. (**A**) Structure-based sequence alignment of PFV and MLV CCD shows the homologous tDNA binding residues of PFV in MLV. Residues 117–252 for MLV CCD IN, 117–252 for PFV CCD IN and 56–182 for HIV CCD IN are displayed. T163 in PFV corresponds to A160 in MLV, Q186 in PFV corresponds to N185 in MLV, A188 in PFV corresponds to P187 in MLV, S193 in PFV corresponds to K192 in MLV and Y212 in PFV corresponds to Y211 in MLV. PROMALS3D structure-based sequence alignment is displayed using the ESPRIPT server (35). (**B**) Residues 117–271 of MLV CCD IN were modeled using three different servers as mentioned in the ‘Materials and Methods’ section. An overlay of the three MLV CCD INs with PFV CCD IN (PDB ID: 3OS1) is presented. The alpha helices are represented in red, the beta sheets are represented in yellow and the loops are represented in green. (**C**) PFV CCD along with the three homology models of MLV CCD is displayed. The tDNA double helix is represented in yellow and was obtained from the PDB ID: 3OS1. tDNA binding residues (blue side chain) of PFV CCD overlaid with the MLV CCD residues predicted to bind tDNA. There is overall agreement in the orientation of the tDNA residue side chains among all the models. The five homologous tDNA binding residues in MLV CCD are A160, N185, P187, K192 and Y211. (**D**) View of the active site of IN is shown in this figure. The secondary structural elements are colored according to the scheme as in (A). The side chain of the active site residues of PFV CCD (light teal), SWISS-MODEL MLV CCD (light magenta), ITASSER MLV CCD (light blue) and MMM-tree MLV CCD (light orange) is aligned to show the conservation of their architecture. The catalytic triad of D125, D184 and E221 of each of the structures are shown aligned in the presence of a single Mg^+2^ ion (green sphere) of the PFV IN structure (PDB ID: 3OS1).

The MLV and PFV IN CCDs have approximately a 30% sequence similarity (Figure 3A). Using a sequence alignment based approach for modeling, we obtained structures with markedly different active site architectures and markedly different spatial organization of homologous tDNA binding residue sites (data not shown). Rather than relying on automated homology modeling servers that build models based on optimal sequence alignment alone, we opted to use a defined template structure with tDNA, specifically the PFV IN target capture complex (PDB ID: 3OS1). Modeling of the MLV IN CCD was performed using multiple servers in order to improve the confidence of our final model structure. The SWISS-MODEL, MMM-tree and I-TASSER servers allow the use of a user-specified template for modeling. Models generated from the SWISS-MODEL, MMMtree and I-TASSER servers are in close agreement with the template PDB ID: 3OS1 structure with respect to side chain organization and overall conservation of secondary structural elements (Figure 3B and C). Of the three models, the I-TASSER model deviates the most from the other models and from the PFV IN CCD structure with respect to active site architecture (Figure 3D).

The final structures from each of the models were evaluated for quality assessment using Verify3D (57), Prosa II (58), Procheck (59) and Molprobity clash scores (60). A summary of these structure validation statistics for the various homology models, obtained using PSVS (31,46), is presented in Supplementary Table S1. The Cα RMSD of the MLV CCD IN models generated by each of the three servers to the PFV CCD structure (PDB ID: 3OS1) is less than 0.8 Å based on DaliLite's pairwise alignment server (55), indicating a high degree of overlap in the various secondary structure motifs, including the D, D (35) E active site residues, and backbone organization.

### Identification of putative tDNA binding residues

Combining the information provided by our solution NMR structure of the MLV IN CTD with our homology models of the MLV IN CCD enabled us to predict regions that potentially interact with tDNA (15). Three predominant features of the MLV IN CTD are initially visible, namely, the presence of a long unstructured tail at the C-terminus of MLV encoding residues 382–408, the positioning of the loop between β1 and β2, and the short length of the β4 strand. In the case of PFV IN, the C-terminal 18 amino acids are not visible in the crystal structure of the inner dimer. For further study, we focused on three regions of MLV IN: the α2 helix within the CCD, the β1-β2 loop of the CTD SH3 fold and the β4 strand of the CTD SH3 domain. The effect that mutating other positively charged residues or residues predicted to bind tDNA had on IN function was also examined. The β1-β2 loop of PFV and MLV bear limited sequence identities in the region expected to interact with tDNA (Figure 2A). The orientation differences in the loops may reflect the presence of the tDNA and/or the influence of other domains of IN. Within the β4 strand, there is no sequence homology between PFV and MLV (Figure 2A). Curiously, the length of the β3 and β4 strands of the PFV CTD (PDB ID: 3OS1), HIV CTD (PDB ID: 1IHV) and the RSV CTD (PDB ID:1C1A) range from five to seven residues as opposed to the four and two residues of the β3 and β4 strands respectively of MLV CTD. The MLV CTD has a longer six residue loop between the β3 and β4 strands, increasing the apparent complexity of this region (Figure 2A and data not shown).

### Virus bearing predicted IN CTD and CCD tDNA binding site mutants affect viability of virus propagation in tissue culture

**Figure 4.**
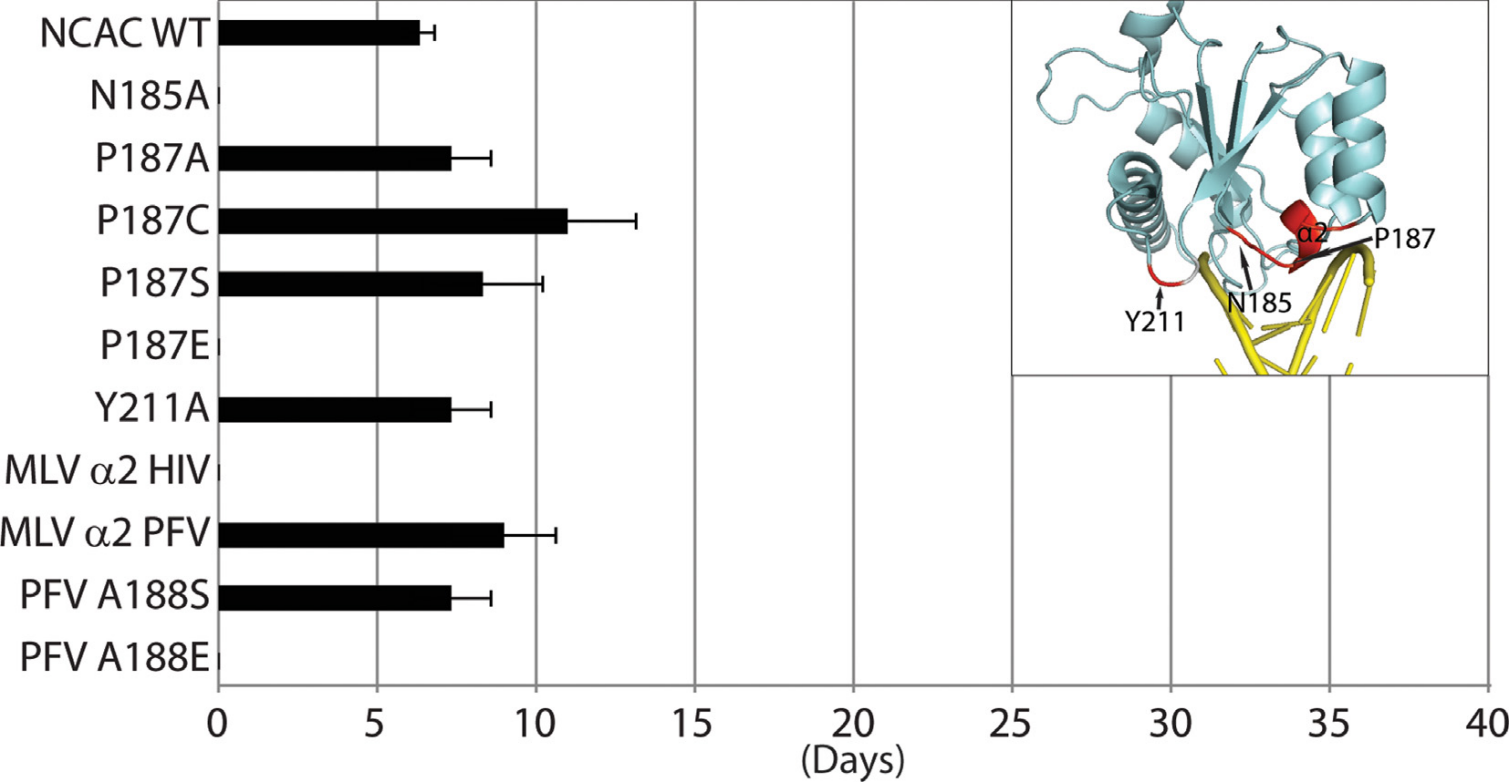
Replication kinetics of MLV IN CCD mutant viruses. The plot represents viral passage of the IN CCD mutant viruses in D17pJET cells measured by RT activity and scored for the day the culture was RT-positive. Standard error bars are indicated (n = 3). Chi-square test was performed to compare the kinetics of the various mutants with that of NCAC WT. Insert highlights the positions of interest on the structure of IN subdomain.

**Figure 5.**
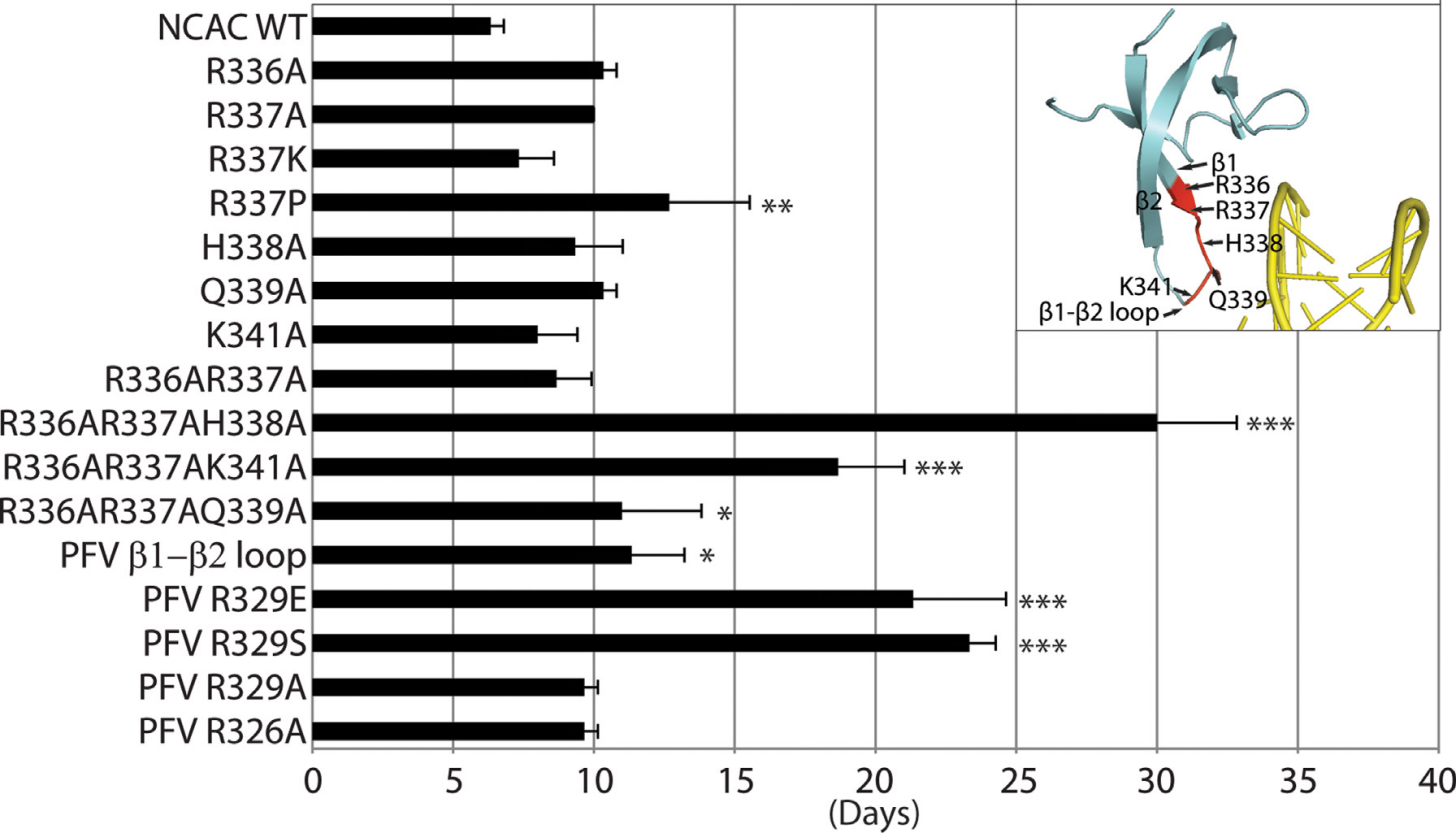
Replication kinetics of MLV IN CTD mutant viruses. The plot represents viral passage of the IN CTD mutant viruses in D17pJET cells measured by RT activity and scored for the day the culture was RT-positive. Standard error bars are indicated (n = 3). Chi-square test was performed to compare the kinetics of the various mutants with that of NCAC WT. Asterisks denote statistically significant differences from NCAC WT; *P < 0.05, **P < 0.01, ***P < 10^−4^. Insert highlights the positions of interest on the structure of IN subdomain.

Within the crystal structure of the PFV IN in complex with tDNA, the α2 helix of the CCD, the β1-β2 loop of the SH3 fold and the β4 strand of the SH3 fold have determinants that bind tDNA (15). We have generated a series of mutants in the homologous region of MLV IN mainly in the CCD α2 helix, CTD β1 strand, CTD β1-β2 loop and the β4 strand of the SH3 fold. Chimeric viruses were also generated, exchanging the PFV α2 helix, HIV α2 helix, the PFV β1-β2 loop, the PFV β4 strand, or the entire SH3 domain of PFV into MLV IN. Figures 4 and 5 and Supplementary Figure S4 summarize all of the mutants that were tested for their effect on virus viability.

A ‘PROMALS3D’ structure-based sequence alignment (34) of the PFV CCD, HIV CCD and MLV CCD SWISS-MODEL is shown in Figure 3A. Previous analysis of PFV indicated the need for amino acids with short side chains at residue A188 as this residue was important in mediating nucleotide base contacts (15). Residues _187_PAFV_190_, _119_SNFT_122_, and _188_AAFT_191_ represent the CCD α2 helix of MLV, HIV and PFV, respectively (Figure 3A). Replacement of the MLV _187_PAFVS_191_ sequence with the HIV _119_SNFTG_123_ sequence rendered the virus non-viable, whereas replacing this sequence with the PFV sequence _188_AAFTS_192_ yielded viable virus with near wild-type (WT) kinetics (Figure 4). To further analyze the importance of the size of the side chain at residue A188 within the MLV-PFV α2 helix chimera, _188_AAFTS_192_ was mutated to either _188_SAFTS_192_ or _188_EAFTS_192_. Consistent with what has been reported for PFV, the sequence _188_SAFTS_192_ was tolerated within the context of the MLV IN (15), whereas the _188_EAFTS_192_ sequence was not (Figure 4). PFV IN A188 is homologous to P187 in MLV IN. MLV IN P187 in the WT viral construct was also mutated to A, E, S and C, where only the P187E was found non-viable (Figure 4). Interestingly, the ovine lentivirus Visna virus, like MLV, also has a P residue in this tDNA binding position (61). Thus, in the context of either the PFV or MLV α2, substituting with an amino acid with a large side chain such as glutamate rendered the virus incapable of propagation in tissue culture.

Since the introduction of the HIV _119_SNFTG_123_ sequence in the CCD α2 helix region was not tolerated, the effect of introducing N at the second position was also examined. MLV IN bearing the point mutant A188N was not viable (Supplementary Figure S4). All three viruses contain a conserved F residue within the α2 helix of the CCD. Virus bearing the F189A substitution in MLV IN was non-viable and thus the F residue appears to be important for maintaining the architecture of the CCD α2 helix (Supplementary Figure S4). In PFV IN CCD; T163, Q186, S193 and Y212 also interact with tDNA making stabilizing contacts. Of these mutant viruses, only MLV IN N185A did not replicate; this position is adjacent to D184, which is part of the active site catalytic triad, and has a deleterious effect on virus propagation (Figure 4 and Supplementary Figure S4).

Within the PFV IN CTD region, the β1-β2 loop, residue R329 in particular, was identified as a critical contact with tDNA (15). R329 induces tDNA bending, enabling access to the catalytic sites in IN that mediate the strand transfer reaction. Exchange of the six residues including the tDNA binding R329 of PFV IN into MLV (_337_RHQTKN_342_ to _327_VARPAS_332_) near the β1-β2 loop resulted in virus capable of propagation in tissue culture. Although R329 is important for PFV tDNA association, mutating this residue to A within the PFV/MLV IN chimera did not affect viral replication. The chimeric virus bearing the VAEPAS PFV β1-β2 loop substitution, however, showed a delay in replication (by 15 days compared to WT) (Figure 5), implying that in the context of the chimeric virus, a negative charge is detrimental. Surprisingly, the chimeric virus bearing the VASPAS PFV β1-β2 loop substitution also showed a delay in replication similar to virus bearing the VAEPAS PFV substitution (Figure 5). In the case of PFV IN, R329S substitution does not affect strand transfer activity, while the R329E substitution drastically affects this activity (15).

Residue R362 of PFV, which makes backbone contacts with tDNA, does not have an apparent homologous equivalent basic residue in MLV strictly based on sequence or structural homology. By side chain organization, the MLV IN R337 in the β1 strand could compensate for PFV IN R362. The role of a positively charged or DNA intercalating amino acid (62) at residue R337 was studied to assess if this residue could potentially interact with DNA. Virus bearing IN R337K replicated similarly to WT MLV, whereas R337P showed an approximately 7 day delay in replication (Figure 5). MLV Q339 structurally aligned with the PFV IN tDNA binding residue R329 (Figure 2A and data not shown). Individual point mutants including R336, R337, H338, Q339 and K341 did not affect viral replication. However, virus bearing the triple mutant, R336AR337K341A, was delayed by about 2 weeks, and virus bearing R336AR337AH338A was severely delayed (by 24 days compared to WT) (Figure 5), and viral titers (LacZ units/ml) (12) decreasing about 10-fold (data not shown). These results imply a redundancy in function of the charged amino acids within the MLV IN β1-β2 loop region. Interaction with the DNA was examined using sequence LOGO analysis (see below).

The β3 and β4 strands and the β3-β4 loop of MLV IN CTD show low homology to PFV and HIV IN CTDs. We generated and analyzed a multitude of point mutants and motif interchanges including exchanging the entire CTD of MLV IN with that of the CTD of PFV IN (Supplementary Figure S4). The majority of the tested mutant viruses, including the chimeras involving the β4 strand, were replication incompetent. The hydrophobic residue W369 was found to be important as only IN W369Y mutant was replication competent, albeit with much slower replication kinetics (Supplementary Figure S4). Additional studies of IN H371 (β4 strand) indicated that viruses bearing IN H371A and H371R were viable; however, substitution of an amino acid (D) with negative charge blocked viral passage (Supplementary Figure S4). In contrast, virus tolerated a D, R or an A substitution at H374 within the 3_10_ helical turn between β4 and β5.

**Figure 6.**
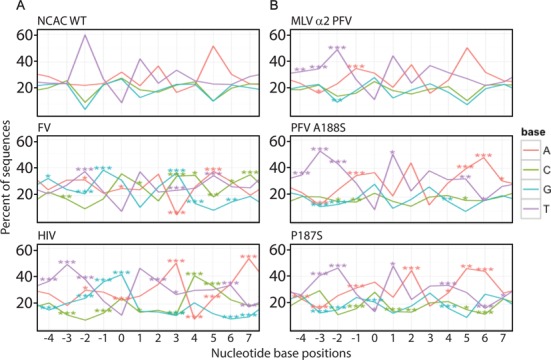
Sequence LOGOs of MLV IN CCD. Sequence preferences at the site of integration were analyzed using an in-house LOGOs program. Datasets used in the study are listed in Supplementary Table S2. Insert box defines the color associated with each nucleotide. Percent of each nucleotide at each position is indicated relative to the scissile bond at position 0. Statistical significances were compared with the NCAC WT dataset using Fisher's exact test, and *P*-values were adjusted using Bonferroni correction at the sample level for multiple comparisons (*P < 0.05, **P < 0.01 and ***P < 0.001). Panel **A** includes MLV WT, FV and HIV datasets, while Panel **B** includes MLV IN CCD mutant datasets.

**Figure 7.**
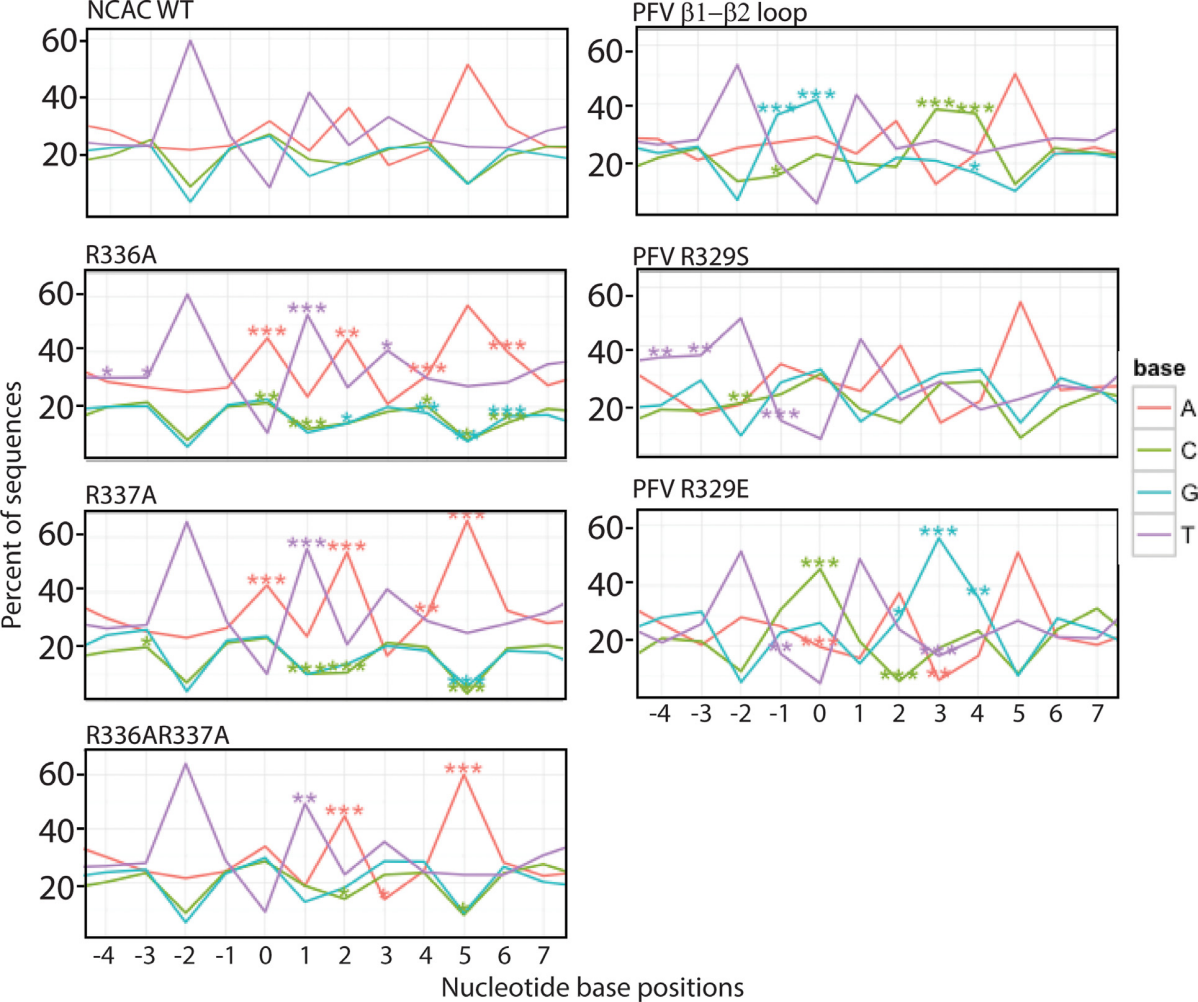
Sequence LOGOs of MLV IN CTD. Sequence preferences at the site of integration were analyzed using an in-house LOGOs program. Datasets used in the study are listed in Supplementary Table S2. Insert box defines the color associated with each nucleotide. Percent of each nucleotide at each position is indicated relative to the scissile bond at position 0. Statistical significances were compared with the NCAC WT dataset using Fisher's exact test, and *P*-values were adjusted using Bonferroni correction at the sample level for multiple comparisons (*P < 0.05, **P < 0.01 and ***P < 0.001).

All viruses that were viable for propagation in tissue culture were sequenced and no second-site mutations or reversions were detected within the IN coding region. Viral Gag polyprotein processing for all the viable mutant proviral constructs appeared similar to that of the WT virus. Mutants that were clustered near the β4 strand showed minor defects with respect to polyprotein processing and stability of the reverse transcriptase (RT) and IN proteins (Supplementary Figure S5). qPCR of minus-strand strong stop (MSSS) and plus-strand extension (PSE) replication intermediates varied between 2- and 7-fold lower for all the defective mutants described (data not shown) in a single-round of infection (12); the MLV IN PFV SH3 showed ∼30-fold decrease in MSSS and ∼23-fold decrease in PSE (12). Analysis of integration site sequences was performed by next-generation sequencing and was limited to replication competent viruses (Figures 6 and 7 and Supplementary Figure S6).

### MLV IN CCD α2 region alters sequence preferences outside the TSD

In order to correlate the function of the α2 helix and the loop between the β1 and β2 strands with tDNA binding, we performed deep sequencing of host-proviral DNA junctions from infected HEK293mCAT genomic DNA. MLV, PFV and HIV display distinct local sequence preferences at the site of integration (63,64). Nomenclature of nucleotide positions has been written using standard International Union of Biochemistry base codes. The generation of viable virus with both chimeric exchanges and point mutants greatly facilitates the understanding of the role of these specific amino acids in target-site binding. The local sequence preferences of the WT virus were compared with HIV and foamy virus integration datasets (Figure 6A). Supplementary Table S2 provides a list of unique integration sites mapped for each mutant.

In MLV, the WT local palindromic sequence preference is _-2_TN↓(V/A)TAT↑NA_+5_ (N refers to any nucleotide and V refers to all nucleotides except T; position 0 is dually coded as V and A), where integration occurs at the 0 and +3 position (in the complementary strand) (Figure 6A and Supplementary Table S3). For PFV, the sequence at 0 and +3 is biased toward G/C (S), at -1 and +4 is biased toward G and C, respectively, and at -2 and +5 is biased toward T/A (W) (_-2_WG↓SNNS↑CW_+5_) (15). The PFV A188S mutant alters the sequence preference to _-3_WNN↓SNNS↑NNW_+6_, expanding the footprint to the -3 and +6 positions (15). Structure-based sequence alignment indicates that MLV IN P187 is homologous to A188 of PFV and S119 of HIV, respectively, with both PFV IN A188 and HIV IN S119 reported to affect target site preference (15,18). Remarkably, the MLV IN P187S mutant shows a similar shift expanding the sequence bias to the -3 and +6 position, with a sequence LOGO of _-3_TT(W/A)↓ATAT↑(T/W)AA_+6_ (Figure 6B and Supplementary Table S3). This expansion of the outer footprint was reproduced in the MLV/PFV chimera within the CCD α2 region (_188_AAFTS_192_). In this chimera, the sequence bias within the TSD remained characteristically MLV (_-3_TTA↓(V/A)TAT↑(W/T)A_+5_). This effect was greatly enhanced when the PFV IN A188S was inserted within MLV (_188_SAFTS_192_), with the sequence LOGO of _-3_TTA↓(V/A)TAT↑(W/T)AA_+6_ and the strong bias for the T/A at the -3 and +6 positions (Figure 6B and Supplementary Table S3). The PFV α2 helical region is proposed to be in close contact with the minor groove of the tDNA. These results indicate that the IN CCD α2 region is contacting sequences flanking the TSD.

### Nucleotide base contact mediated tDNA binding in MLV IN CTD occurs through β1-β2 loop residues

To examine the role of the MLV IN CTD β1-β2 loop in target site selection, we substituted six residues of the MLV IN (_336_RRHQTKN_342_) with that of the PFV IN (_326_RVARPAS_332_). Substitution of the PFV VARPAS into MLV IN changed the base preference closer to that of PFV IN (_-2_TG↓GTAC↑CA_+5_) (Figure 7 and Supplementary Table S3). The positions -1, 0, +3 and +4 now show a strong preference for G and C nucleotides. This indicates that the loop region is the main determinant for tDNA binding at the position of the scissile bond. In PFV IN, a single R329 residue mediates extensive base contacts and engages in hydrogen bond interactions with both strands of tDNA. This is responsible for inducing a sharp bend making the tDNA more amenable for strand transfer. R231 is the corresponding amino acid in HIV IN that appears to be carrying out a similar role (18). Sequence LOGOS analysis of PFV IN R329S resulted in the loss of bias at positions 0 and +3, with equal distribution of CAG and GTC at these positions, respectively (15). PFV IN R329E, in contrast displayed a strong enhancement for C at position -1 and 0 and G at +3 and +4 (15). Incorporation of these point mutants within the PFV β1-β2 chimera (VASPAS and VAEPAS) paralleled these studies, with the MLV/PFV IN VASPAS (IN PFV R329S) now displaying the LOGO _-2_TV↓VTAB↑SA_+5_ and the IN VAEPAS (IN PFV R329E) the LOGO _-2_TC↓CTAG↑GA_+5_ (Figure 7 and Supplementary Table S3) (B refers to all nucleotides except A). Thus, the substitution of the PFV IN CTD β1-β2 loop within MLV IN recapitulates the specificity defined for PFV, in particular as it relates to PFV IN R329.

**Figure 8.**
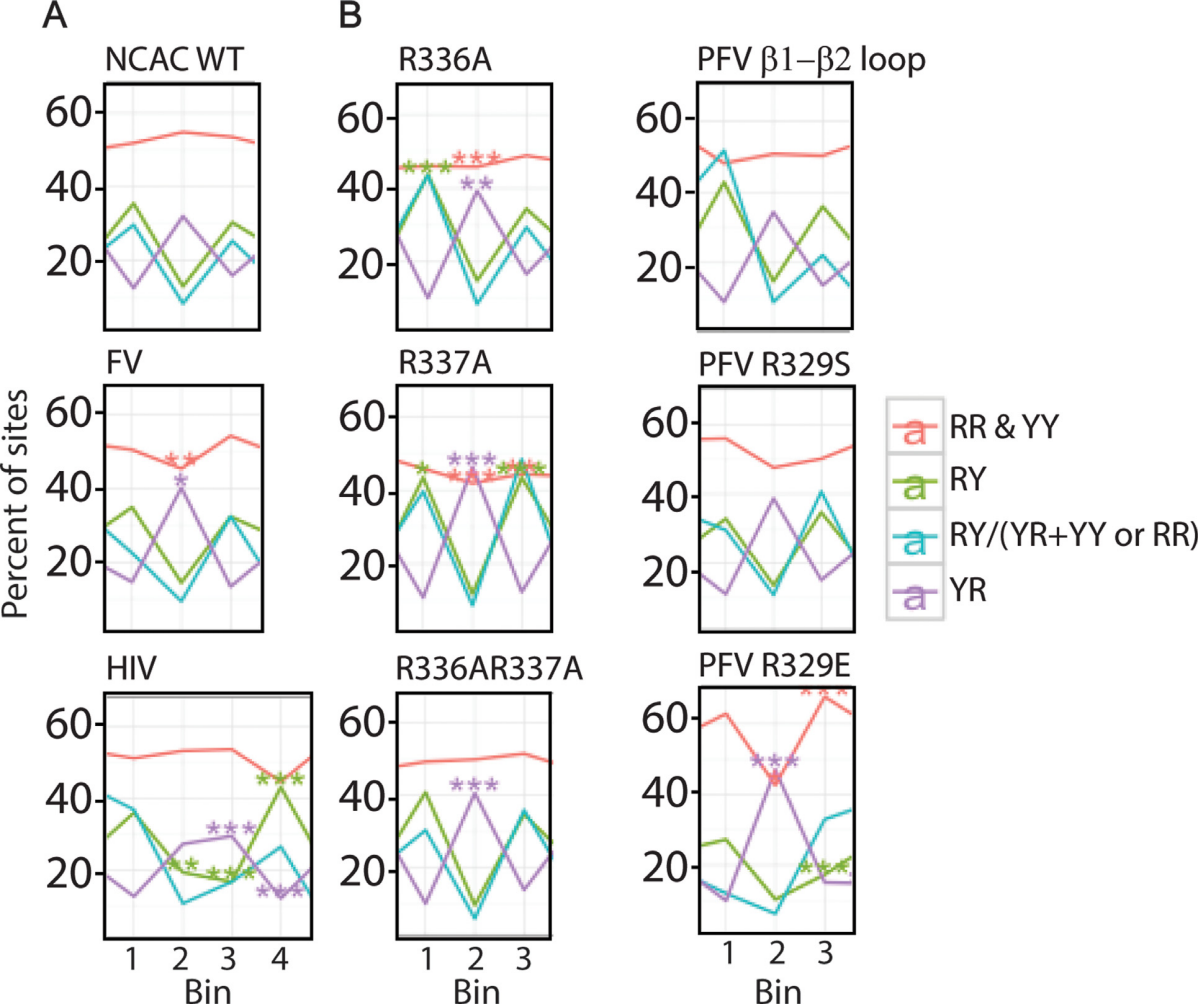
Dinucleotide flexibility in the region of TSD. (**A**) MLV and FV have a 4-bp TSD and therefore three dinucleotide steps of 0–1, 1–2 and 2–3 marked as 1, 2 and 3 in the bottom. Since HIV has a 5-bp TSD, four stands for 3–4. (**B**) MLV IN CTD β1-β2 loop mutants are shown with respect to flexibility preferences at TSD. For both panels, red line denotes purine–purine (RR) and pyrimidine–pyrimidine (YY) (intermediate flexibility), green line denotes purine–pyrimidine (RY) (rigid), violet line denotes pyrimidine–purine (YR) (flexible) and turquoise line denotes ratio of rigid dinucleotide steps to flexible and intermediate flexible dinucleotide steps. Analysis was performed as described in (18) and in addition the *P*-values were subjected to Bonferroni correction for multiple comparisons.

Studies were performed to analyze the role of specific MLV IN amino acids in binding the tDNA with respect to flexibility of nucleotide bases at the site of integration. Flexibility of bases has been proposed to be important for the binding mechanism of IN to tDNA. Pyrimidine–pyrimidine and purine–purine dinucleotide steps show intermediate flexibility, while purine–pyrimidine dinucleotide shows the least flexibility, and pyrimidine–purine dinucleotide shows the most flexibility. The flexibility of dinucleotide steps is in turn a function of nucleotide base stacking properties (18). Unlike PFV and MLV IN that show four base pair TSDs, HIV IN shows five base pair TSDs and therefore four continuous dinucleotide steps. In the case of HIV IN, the tDNA base preference shows a flexible central dinucleotide preference flanked with rigid dinucleotide steps within the TSD region (18). For WT MLV IN, wherein the TSD region consists of four nucleotides and therefore three dinucleotide steps, the flanking dinucleotide steps also show rigidity, while the central dinucleotide step shows flexibility (Figure 8A).

A structural alignment of an ensemble of MLV IN CTD structures with that of PFV IN CTD in complex with tDNA shows that R337 and K341 are in close proximity to tDNA (Figure 2C). Of particular interest is the arginine dipeptide (R336R337) in the β1-β2 loop region. LOGO analysis of either IN R336A or R337A results in a distinct target site preference. The strong bias for the sequence _-2_TW↓ATAT↑WA_5_ is statistically enhanced over WT at almost every position. This sequence results in a highly flexible dinucleotide central TSD (TA) surrounded by rigid dinucleotide positions (PuPy). The flexible structure provided by this target site is preferred by IN proteins mutated at either R336 or R337, implying a functional redundancy for these basic residues. Substitution of both IN R336 and R337 to A maintains the bias for the flexible core and rigid flanking TSD sequence (Figure 8B). But unlike either of the single mutants, the double mutant mimics the WT virus for positions 0 and +3 for sequence preference (Figure 7). The effects at the central core of the TSD does not imply direct interaction with R336 or R337, rather the flexibility permits the positioning and bending of the target site within the intasome structure allowing accessibility of the viral DNA for nucleophilic attack. The chimeric virus bearing the VASPAS (IN PFV R329S) sequence showed a dinucleotide preference pattern similar to WT, but strikingly, the VAEPAS sequence (IN PFV R329E) showed a relatively greater requirement for central flexible dinucleotide and flanking intermediate rigid dinucleotide at the TSD. Unlike WT virus, the preference for rigid dinucleotides was far lower in the case of VAEPAS (Figure 8B).

Unlike PFV IN, where R329 plays a critical role in interacting with both strands of the TSD, no one amino acid within the MLV IN β1-β2 loop appears to dominate target-site recognition (Supplementary Figure S6). Triple mutants of IN R336AR337A with H338A, Q339A or K341A show the characteristic bias for the TA flexible dinucleotide within the TSD observed for R336AR337A. However, it should be noted that virus bearing the IN triple mutants R336AR337AH338A and the IN R336AR337AK341A but not R336AR337AQ339A replicated significantly slower than WT (Figure 5), indicating that substitutions of multiple amino acids involved in binding the host DNA do affect the overall fitness of the virus. Supplementary Table S3 summarizes the mutants tested and their effect on sequence recognition.

The primary DNA preferences can have pleiotropic effects beyond dinucleotide flexibility on parameters including the global targeting profile preferences. The strong bias of mutant virus bearing IN R336A and R337A (within the CTD) and CCD changes in the IN α2 region (IN P187S, PFV A188S) for AT sequences was in fact reflected in an increased bias against GC rich regions and a preference for DNase I hypersensitive sites compared to the WT virus (Supplementary Figure S7A). These changes were observed with distances as far as 100 K base pairs away from the site of integration. Similarly, virus bearing IN PFV R329E, which increased local sequence bias for GC content, showed related increases for integration into G/C dense regions.

Disrupting the interaction between host BET proteins and MLV IN through deletion of the IN C-terminus (INΔC) was shown to reduce integration frequency near transcription start sites (TSS) and CpG islands (12) due to the association of BET binding sites at promoter regions. Surprisingly, global targeting to CpG islands and TSS was altered in several IN mutant viruses that maintained the WT local target sequence preferences (Supplemental Table S3). These include virus bearing the double mutant IN R336A/R337A, which also shows an increased bias for dinucleotide flexibility (TA) at the central position of the TSD (Figure 8 and Supplemental Figure S7A), and triple mutant combinations including these residues (IN R336A/R337A/H338 and IN R336A/R337A/K341A) (Supplemental Figure S7A and B). Chimeric mutant IN PFV R329S also shows decreased frequency of integration near CpG islands (Supplemental Figure S7A). These reduced propensities for integration near TSS and CpG islands, however, do not parallel the effect observed in the MLV INΔC mutant that affects the BET protein-IN interaction (12) (Supplementary Figure S7B). Thus, mutants affecting tDNA recognition also affect integration patterns near specific genomic features.

## DISCUSSION

Here we present a study of MLV IN domain structures and use of the structures to identify tDNA binding residues. We report the comprehensive analysis and validation of the solution NMR structure of the MLV IN CTD, which is consistent with other retroviral CTD domains in adopting an SH3 fold. Homology modeling and structural analysis of the MLV IN CCD was greatly assisted by the available PFV IN intasome coordinates (15), which we used as template. Through structure-based sequence alignment, the MLV IN CTD construct was modified from initiating at residue IN 287 to IN 329, resulting in an MLV construct which was more suitable for NMR analysis. Monomeric MLV IN CTD is consistent with what is predicted based on the crystal structure of the PFV IN where the individual CTDs of the tetramer do not form intermolecular CTD–CTD contacts (6). However, it is interesting to note that the SAXS analysis of the ASV IN and HIV-1 IN revealed potentially important CTD–CTD interactions as part of a ‘reaching dimer’ (65,66). These interactions could result in the formation of an alternative conformation to the PFV IN CCD–CCD dimer interaction. Although the SAXS data were obtained in a structure devoid of tDNA or viral LTR (65,66), the SAXS data of PFV IN suggest that in the presence of viral DNA, the outer CTDs do not appear to make stable interactions with the inner CTDs (16), indicating that the organization of the IN CTD in lentiviruses and alpharetroviruses might function differently than gammaretroviruses and spumaretroviruses.

The structure of the MLV IN CTD is unique in that it includes the complete sequence through the terminus of IN (residue 408). Although the C terminal tail is largely disordered from residues G382 to P408, there are a few transient Nuclear Overhauser Effects, indicating the formation of transient structural motifs (data not shown). The elucidation of the complete CTD structure, including the disordered tail region, has enabled us to recently show that the C-terminal tail subsequent to G382 undergoes a disorder-to-order transition in the presence of host BET proteins Brd2, 3 and 4 (12). This interaction is important for dictating the target site preference of transcription start sites, CpG islands and possibly active enhancers for MLV integration in host cells (3–4,12,14). Deletion of 28 amino-acid residues and linker insertion between the 30th and 31st amino-acid residue from the C-terminus were tolerated in tissue culture, while truncation of 31 and 34 amino acids rendered the virus incapable of spreading in tissue culture (51,67). The INΔ34 virus is also reported to affect the level of viral reverse transcription (68). The biochemical and genetic data match exquisitely with the solution structure of the IN CTD. The terminus of the IN CTD β5 corresponds with the position defined for the deletion of the terminal 31 amino acids. Truncations beyond this would disrupt the SH3 fold as defined by the MLV IN CTD structure.

The C-terminal tail region of MLV IN CTD is not required for viral propagation in tissue culture (51,67) or *in vitro* integration activity (12,69); however, it plays a role in the interaction with host BET proteins, and it is possible that this region has additional functions. In PFV, the last amino-acid residue within the CTD that is structured (H374), traverses through the structure to contact the viral LTR. The C-terminal tail is rich in basic amino-acid residues, which could result in non-specific binding of DNA. Progressively truncating the IN C-terminus region in HIV showed increasingly delayed replication kinetics as the truncations approach the boundary of the SH3 fold (70,71). C-terminal tail truncations present a Class II mutant phenotype in which the non-enzymatic activities of IN as well as the activity of reverse transcriptase are affected (71). A more thorough truncation analysis of the HIV-1 IN C terminus by Mohammed *et al*. (70) shows that 3′ processing activity of IN is also affected with these truncations, but strand transfer is not, indicating that the tail region may serve to stabilize the viral LTR during the 3′ processing reaction.

Gammaretroviruses and spumaretroviruses are phylogenetically more closely related to each other than to lentiviruses (72). The INs of these viruses also differ in their interactions with host or viral proteins and in their target site preferences (2,73–78). Maertens *et al*. have shown that the WT PFV IN has a weak preference for symmetric bases near the site of integration (15). In this study, the weak symmetric base preference at the site of integration was observed in all of the MLV IN WT and viable mutant viruses that were analyzed.

**Figure 9.**
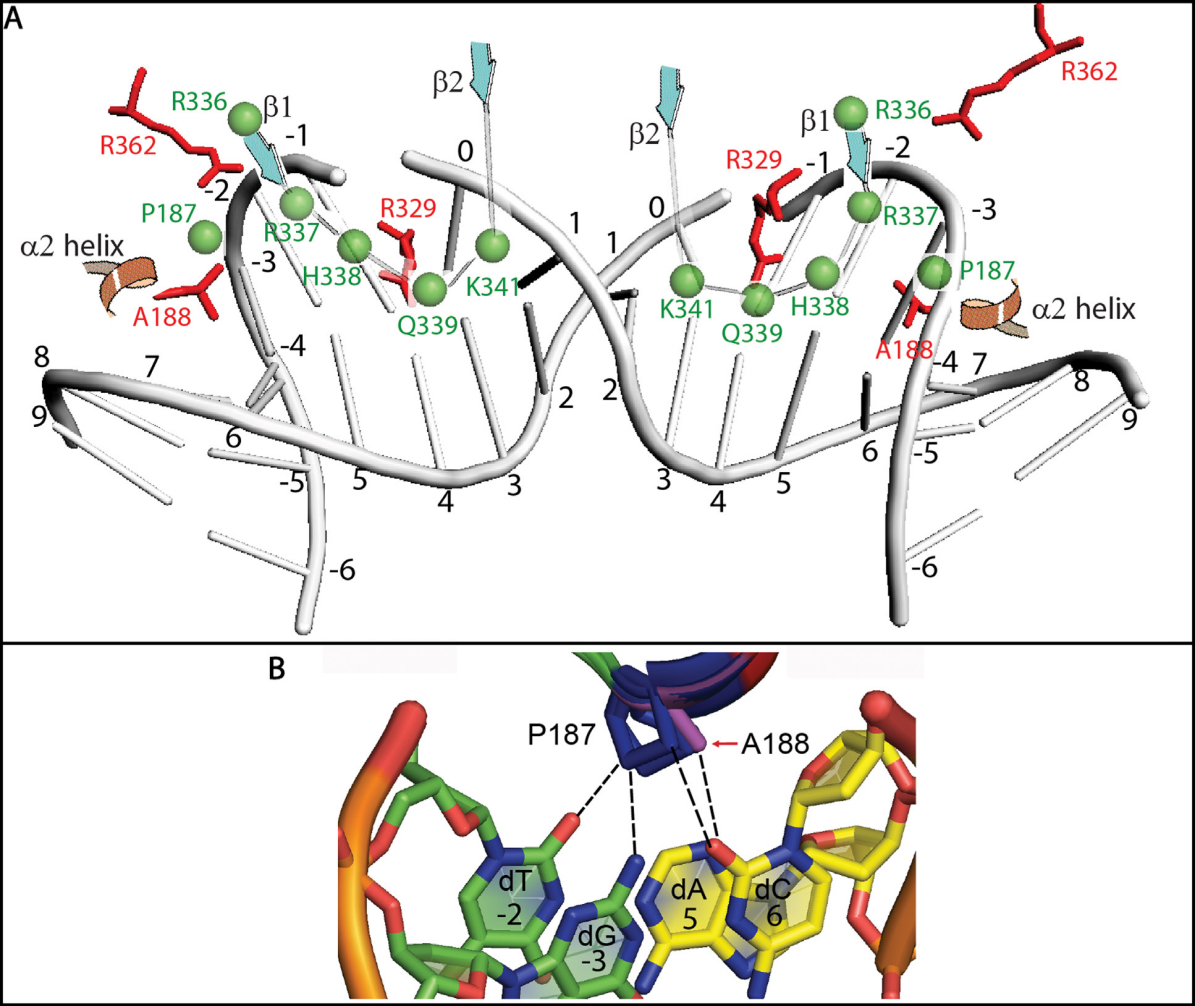
Model for MLV IN tDNA binding. (**A**) This model represents the orientation of the MLV IN tDNA binding residues relative to the PFV IN counterparts. The positioning of bent DNA was extrapolated from the PFV IN strand transfer complex (3OS0). Numbers from -6 to 9 indicate the position of nucleotide bases relative to the scissile bond position at 0. Green spheres and green labeling indicates MLV IN amino acids. Red sticks and red labeling indicates PFV IN amino acids. CTD β1and β2 strands and CCD α2 helix are indicated. (**B**) Magnified view of panel C is shown in order to focus on the potential interactions mediated by P187. -3 (dG), -2 (dT), 5 (dA) and 6 (dC) positions of tDNA are shown. The DNA bases in yellow (5 and 6 positions) are from one strand, while the bases in green (-3 and -2 positions) are from the complementary strand.

The solution structure of the IN CTD along with the molecular model of the MLV CCD allowed for the identification of specific residues with potential to interact with tDNA. Figure 9A summarizes the putative functions of these IN domains in interacting with tDNA. In these studies, three main regions have been studied using chimeras between MLV with PFV or HIV as well as point mutants. Within the CCD, the α2 helix was identified both structurally (15) and biochemically (61) to be important for tDNA binding. The characteristics of this α2 region in MLV is consistent with these studies, indicating that the α2 region of PFV sequence can replace that of MLV, and that potential tDNA binding amino acids with large side chains sterically interfere with the function of this domain. Chimeric MLV bearing the PFVα2 region displayed a widened LOGO footprint, indicating binding to regions flanking the TSD (Supplementary Table S3 and Figure 6).

Based on molecular models of the CCD and 454 sequencing data, P187 in the MLV IN CCD can make nucleotide base contacts and can functionally mimic the role of A188 of PFV IN and S119 of HIV IN (Figure 9B). Prolines in general have a higher tendency to form van der Waal's contacts with adenosine and to a lesser extent thymidine (79); however, A188 in PFV IN makes contacts with position +6 of the tDNA (15). There is a strong preference for T and A in the -2 and +5 positions of the MLV IN WT sequence preference, indicating the possibility that P187 may interact with those positions. Indeed, an overlay of the CCD structural model shows that the orientation of P187 allows it to be in close proximity to the -2, -3 and +6 positions of tDNA.

Despite a lack of sequence identity, the amino-acid residues within the PFV IN CTD β1-β2 loop important for tDNA binding can functionally replace the equivalent region of MLV. It has been previously shown that mutations in the region encoding this loop of PFV IN result in the retention of IN-EGFP fusion proteins within the cytoplasm, indicating a role for this region in the karyophilic property of PFV IN (80). For PFV IN, R329 has hydrogen bonding to bases in both strands of the TSD. HIV IN has a conserved arginine in that position, whereas in MLV IN the homologous amino acid is a glutamine. However, a structure-based alignment of the MLV IN CTD with the PFV IN strand transfer complex shows proximity of R337 and K341 near tDNA. For MLV IN, no one amino acid was identified as essential, with combinatorial effects observed for mutants of IN R336/R337 with H338, K341 or Q339. The β1-β2 loop as a whole might function to stabilize tDNA binding and bending. LOGO sequencing analysis indicated similar requirements for sequence/flexibility for viruses mutated at either basic residues at position R337 or R336, implying a redundancy of function. Based on molecular modeling, the MLV IN R337 residue in the CTD β1 strand can potentially replace the function of the R362 residue in PFV IN (Figure 2B and C). We propose that the R336/R337 residues may function similar to the PFV IN R362 residue which was previously shown to interact with the phosphate backbone at the -2 position and be essential for tDNA binding (15). Furthermore, substitution of a part of the β3-β4 loop and the entire β4 strand of MLV with the PFV β4 strand sequence did not yield infectious virus. There is little primary sequence homology between MLV and PFV, with the β3-β4 loop and β4 strand of MLV consisting of hydrophobic amino acids. IN W369 is essential for viral propagation, although there is no evidence that it is involved in binding tDNA. Similarly, for the three mutants in the β4 strand region that were viable, IN W369Y, H371A and H374A, sequencing data did not indicate any base-specific interaction with tDNA (Supplementary Table S3 and Supplementary Figure S6).

Surprisingly, for MLV IN Y211A, we observed viral replication at near WT kinetics. However, it was previously reported that this mutant compromised concerted two-end integration activity *in vitro*, although disintegration activity was observed. The presence of a hydrophobic residue at position 211 was concluded to be important (81).

In our studies, integration of MLV IN mutants of specific tDNA interacting residues displayed altered global targeting preferences. This is in agreement with recent reports in HIV, where alteration of residues involved in tDNA interactions affected a host of global targeting parameters, independent of LEDGF/p75 mediated targeting (82). Comparison of the integration sites of MLV mutated in the IN tDNA binding sites against a ChIP-seq dataset of BET protein binding sites in HEK293 cells yielded no correlation with total BET protein interactions and a modest effect on BET protein sites near promoter regions (data not shown). However, it is unclear whether this modest decrease in targeting toward TSS and CpG islands for mutants affecting tDNA interactions within the β1-β2 loop (Supplementary Figure S7B) is due to a BET-independent pathway or through secondary stabilization of contacts.

Although traditionally SH3 domains have been observed to be important for protein–protein interaction, in archaebacteria, the SH3 fold within the Sso7d/Sac7d proteins functions as DNA architectural domains (83–85), inducing bending and compaction of the DNA. In PFV IN, R329 is involved in tDNA bending, allowing the phosphodiester bond to be accessible for the divalent cation to mediate strand transfer reaction (15). Also, the outer CTD domains that remain unstructured within the PFV intasome crystal structure could play a more generalized architectural function with the DNA or nucleosomes.

In conclusion, with the aid of the CTD NMR structure, CCD homology model structures, and next-generation sequencing of mutant and WT viruses, we have developed a model for tDNA binding by MLV IN that implicates the α2 helix of the CCD and the β1-β2 loop of the CTD to be important determinants of tDNA binding.

## ACCESSION NUMBER

Sequences were deposited in the sequence read archive (SRA) with accession number SRP04876.

## SUPPLEMENTARY DATA

Supplementary Data are available at NAR Online.
